# Post‐COVID‐Syndrome Patients Might Overestimate Own Cognitive Impairment

**DOI:** 10.1111/ene.70195

**Published:** 2025-05-15

**Authors:** Sofia Wöhrstein, Tamara Matuz, Lilli Rötzer, Hans‐Otto Karnath

**Affiliations:** ^1^ Center of Neurology, Division of Neuropsychology Hertie Institute for Clinical Brain Research, University of Tübingen Tübingen Germany

**Keywords:** COVID‐19, neurocognitive disorder, objective cognitive impairment, post‐COVID syndrome, subjective cognitive complaints

## Abstract

**Background:**

After a COVID‐19 infection, some patients experience long‐term consequences known as Post‐Covid Syndrome, which often includes cognitive impairment. We investigated the congruence between subjectively experienced and objectively measured cognitive deficits after a COVID‐19 infection in an unselected, successively admitted cohort of 46 patients reporting subjective cognitive complaints (SCC).

**Methods:**

We employed a comprehensive neuropsychological test battery to assess objective cognitive impairment across various cognitive domains. Three different cut‐off criteria were applied, commonly used in the literature to define objective neurocognitive disorder (NCD).

**Results:**

We observed a notably low congruence between SCC and NCD in Post‐Covid Syndrome, regardless of the cut‐off criterion. Depending on the cognitive domain, only 4% to maximally 40% of the SCC could be objectified.

**Conclusions:**

One possible explanation for this discrepancy could be the high rate of depressive symptoms observed in the group of patients studied, which may negatively influence the perception of one's cognitive abilities. These findings emphasize the need for careful evaluation of SCC in Post‐Covid Syndrome and suggest that treating depressive symptoms may also alleviate some of the perceived cognitive deficits.

**Abbreviations**
COVID‐19: coronavirus disease 2019
DSM‐5: Diagnostic and Statistical Manual of Mental Disorders, fifth edition
IQR: interquartile range
MMSE: Mini‐Mental State Examination
MoCA: Montreal Cognitive Assessment
NCD: neurocognitive disorder
PCS: Post‐COVID syndrome
PR: percentile rank
SARS‐CoV‐2: severe acute respiratory syndrome coronavirus type 2
SCC: subjective cognitive complaints
SD: standard deviation

## 1 Introduction

Some patients who have been infected with severe acute respiratory syndrome coronavirus type 2 (SARS‐CoV‐2) experience long‐term effects following their infection [1, 2, 3]. The terminology used for this condition varies and includes different terms: for example, Long COVID, Post‐COVID symptoms, Post‐COVID syndrome, Post‐COVID condition, post‐acute COVID [3, 4, 5, 6]. In line with the definition of COVID‐19 long‐term effects proposed by the National Institute for Health and Care Excellence (NICE) [7], which was also incorporated by the latest German S1 guideline for Long‐/Post‐COVID [5], we use the term *Post‐COVID Syndrome* (PCS) in the following to address symptoms that (i) develop during or after COVID‐19 infection, (ii) continue for more than 12 weeks, and (iii) cannot be explained by any other disease or diagnosis.

Around 65 million individuals worldwide are estimated to be affected by PCS, while cases are still increasing [4]. Pathomechanisms that may explain PCS include persistent viral reservoirs, immune dysregulation, autoimmunity and endothelial dysfunction, and chronic inflammation [8, 9, 10]. In addition to the respiratory/pulmonary sequelae, fatigue, decreased exercise tolerance, headache, anxiety, depression, insomnia, and “brain fog” were frequently reported as persistent symptoms [11, 12]. Beyond that, numerous studies have reported cognitive impairments related to PCS [12, 13, 14, 15, 16]. Many of these studies rely on either self‐reported subjective cognitive deficits in various forms or on objective results from short screening instruments such as the Montreal‐Cognitive‐Assessment (MoCA) [17] or the Mini‐Mental‐State‐Examination (MMSE) [18].

A significantly smaller amount of research has focused on characterizing cognitive performance in PCS using comprehensive test batteries and clinical diagnostic criteria. They have identified deficits in attention, memory, and executive functioning following SARS‐CoV‐2 infection [14, 19, 20, 21]. However, the incidence rates of global cognitive impairment or of domain‐specific cognitive deficits fluctuate due to methodological variations regarding data collection, sample characteristics, disease severity, comorbidities, time span between infection and symptoms, and not at least due to different definitions of cognitive impairment and methods of assessment. In a meta‐analysis, Ceban et al. [11] reported that 20% of the population examined in 43 studies showed cognitive impairment 12 or more weeks after COVID‐19 infection. Another systematic review based on 66 studies reported incidence rates ranging from no cognitive impairment to 78% prevalence in at least one cognitive domain [22].

Given these heterogeneous rates of objective cognitive deficits persisting after COVID‐19 infection and the lack of unanimous classification criteria reported to date, the present study (i) analyzes possible differences between subjective complaints and objective test results and (ii) compares the impact of different diagnostic criteria for objective cognitive disorders used in the literature. Understanding the interplay between subjectively perceived deficits in different cognitive domains and objective cognitive performance is crucial for better assessment as well as treatment options and rehabilitative measures, ultimately leading to effective patient support and a better quality of life [23, 24, 25]. To this end, we examined cognitive performance in an unselected, successively admitted group of PCS patients using comprehensive neuropsychological tests. Over a span of more than four years, these patients visited our Center for Neurology at the University Hospital Tübingen, Germany, because of subjectively perceived cognitive complaints.

## 2 Methods

### 2.1 Participants

All patients (*N* = 60) who were referred to the Division of Neuropsychology at the Center of Neurology at Tübingen University Hospital between 10/2020 and 02/2025 for a neuropsychological evaluation of subjectively perceived cognitive deficits (subjective cognitive complaints, SCC) after COVID‐19 infection (diagnosis included PCR and/or rapid tests) were screened. In accordance with the German S1 guideline for PCS [5], they were included in the present study if their SCC (i) persisted for at least 12 weeks after their COVID‐19 infection, (ii) could not be explained by other diagnosed neurological and/or current psychiatric disorders, and (iii) did not occur after COVID‐19 vaccination. This study was approved by the Ethics Committee of the Medical Faculty of the University of Tübingen, Germany (authorization number 233/2024BO2).

### 2.2 Neuropsychological Evaluation

The neuropsychological evaluation was conducted by a trained clinical neuropsychologist (T.M.) and lasted between 90 and 120 min. After a very thorough anamnesis, which served to categorize the SCC, the following neuropsychological tests were performed, all in their German version: verbal learning and memory ability test (Verbaler Lern‐ und Merkfähigkeitstest [VLMT]) [26], Wechsler Memory Scale—Fourth Edition (WMS‐IV) [27], Regensburg word fluency test (Regensburger Wortflüssigkeits‐Test [RWT]) [28], Tower of London (TL‐D) [29], Nürnberg age inventory (Nürnberger‐Alters‐Inventar [NAI]) [30], attention test battery (Testbatterie zur Aufmerksamkeitsprüfung [TAP]) [31], and the Rey‐Osterrieth Complex Figure Test (ROCFT) [32, 33]. Test performance was expressed in percentile ranks according to the respective normative test data (adjusted for age, education, and/or sex). In addition, a depression screening was performed using the long version of the general depression scale (Allgemeine Depressionsskala—lange Fassung [ADS‐L]) [34].

### 2.3 Data Analysis

The percentile ranks from the individual subtests of the neuropsychological test battery were transformed into standardized *z*‐scores using psychometrica [35]; all further analyses were performed using *R Studio* (R version 4.4.0) [36]. Following the Diagnostic and Statistical Manual of Mental Disorders (DSM‐5) [37], objective cognitive impairment, that is, a neurocognitive disorder (NCD), was defined as a *z*‐score below one standard deviation (SD) from the respective *z*‐score mean. If the patient had a *z*‐score below one but less than two SDs below the mean, his/her impairment was categorized as “mild”. If the *z*‐score was lower than two SDs, the impairment was categorized as “major”. Based on the DSM‐5 criteria for NCD, we constructed five cognitive domains from the subtests of the aforementioned neuropsychological test battery: (1) Attention, (2) Memory and Learning, (3) Executive Function, (4) Word Fluency, and (5) Visual Reproduction. Table 1 shows which subtests were assigned to which domains. In parallel, we also categorized patient‐reported symptoms and deficits (SCC) in the same five cognitive domains.

**TABLE 1:** Assignment of (sub‐)tests of the neuropsychological evaluation to cognitive domains.

Domain	Cognitive task	Neuropsychological (sub‐)test
Attention	Divided attention	TAP divided total
	Tonic attention	TAP tonic alertness
	Phasic attention	TAP phasic K value
	Flexible attention	TAP flexible
Memory and Learning	Verbal learning	VLMT sum rounds 1–5/WMS‐IV logical memory
	Verbal recall immediate	VLMT difference round 5‐round6/WMS‐IV logical memory
	Verbal recall delayed	VLMT difference round 5‐round 7/WMS‐IV logical memory
	Verbal recognition	VLMT recognition/WMS‐IV logical memory
	Short term memory	WMS‐IV digit span forward
Executive Function	Working memory	NAI/WMS‐IV digit span backward
	Categorical switch word fluency	RWT sports/fruits
	Categorical switch word fluency	RWT G/R‐words
	Cognitive planning	TL‐D
Word Fluency	Semantic word fluency	RWT animals
	Phonematic word fluency	RWT P‐words
Visual Reproduction	Visual reproduction immediate	ROCFT/WMS‐IV figure
	Visual reproduction delayed	ROCFT/WMS‐IV figure
	Visual recognition	ROCFT/WMS‐IV figure

Abbreviations: NAI, Nürnberg age inventory [27]; ROCFT, Rey‐Osterrieth Complex Figure Test [29, 30]; RWT, Regensburg word fluency test [25]; TAP, attention test battery [28]; TL‐D, Tower of London [26]; VLMT, verbal learning and memory ability test [23]; WMS‐IV, Wechsler Memory Scale—Fourth Edition [24].

To date, various analysis criteria as well as NCD cut‐off scores have been used in the literature to analyze and classify cognitive domains. To compare the effects of using these different approaches, we analyzed our data according to the following three criteria. The first and most liberal criterion “*subtests overall*” (e.g., Schild et al. [14]) refers to the standardized *z*‐scores for each subtest. In this case, if a patient had an impairment in at least two subtests of the total of 18 subtests *across all five cognitive domains* (see above), he/she was classified as having an NCD. A second criterion “*mean domain*” (e.g., Bungenberg et al. [20]) is based on the mean *z*‐score per cognitive domain, that is, the averaged *z*‐score across all subtests that make up a particular cognitive domain. In the third and most conservative criterion “*subtests domain*” (cf. American Psychiatric Association [37]), we considered the individual *z*‐scores for each subtest *per cognitive domain*. If at least two subtests in a domain were between one and two SD below the mean of the subtest, the domain was classified as “mildly” impaired; if two subtests were more than two SD below the mean of the subtest, the domain was classified as “majorly” impaired. If only one subtest was at least one SD below the mean, this domain was classified as “not impaired”. According to the DSM‐5 guidelines, only one of the five cognitive domains had to be impaired to be classified as a mild or major general NCD.

## 3 Results

### 3.1 COVID‐19 Infections Without Hospitalization

#### 3.1.1 Final Patient Sample

The inclusion criteria were met by 44 patients (28 females) aged 19 to 61 years (M = 47.1; SD = 12.3) in whom the COVID‐19 infection did not require hospitalization, as well as two additional female patients aged 42 and 50 years who required hospitalization during their acute COVID‐19 infection. Fourteen patients were excluded (twelve of them experienced a COVID‐19 infection without hospitalization; two with hospitalization). For the non‐hospitalized group of the final patient sample, the mean time between COVID‐19 infection and neuropsychological examination was 17.7 months (SD = 9.8); the two hospitalized patients were examined 11 and 16 months after their respective COVID‐19 infections.

#### 3.1.2 Subjective Cognitive Complaints (SCC)

**FIGURE 1:**
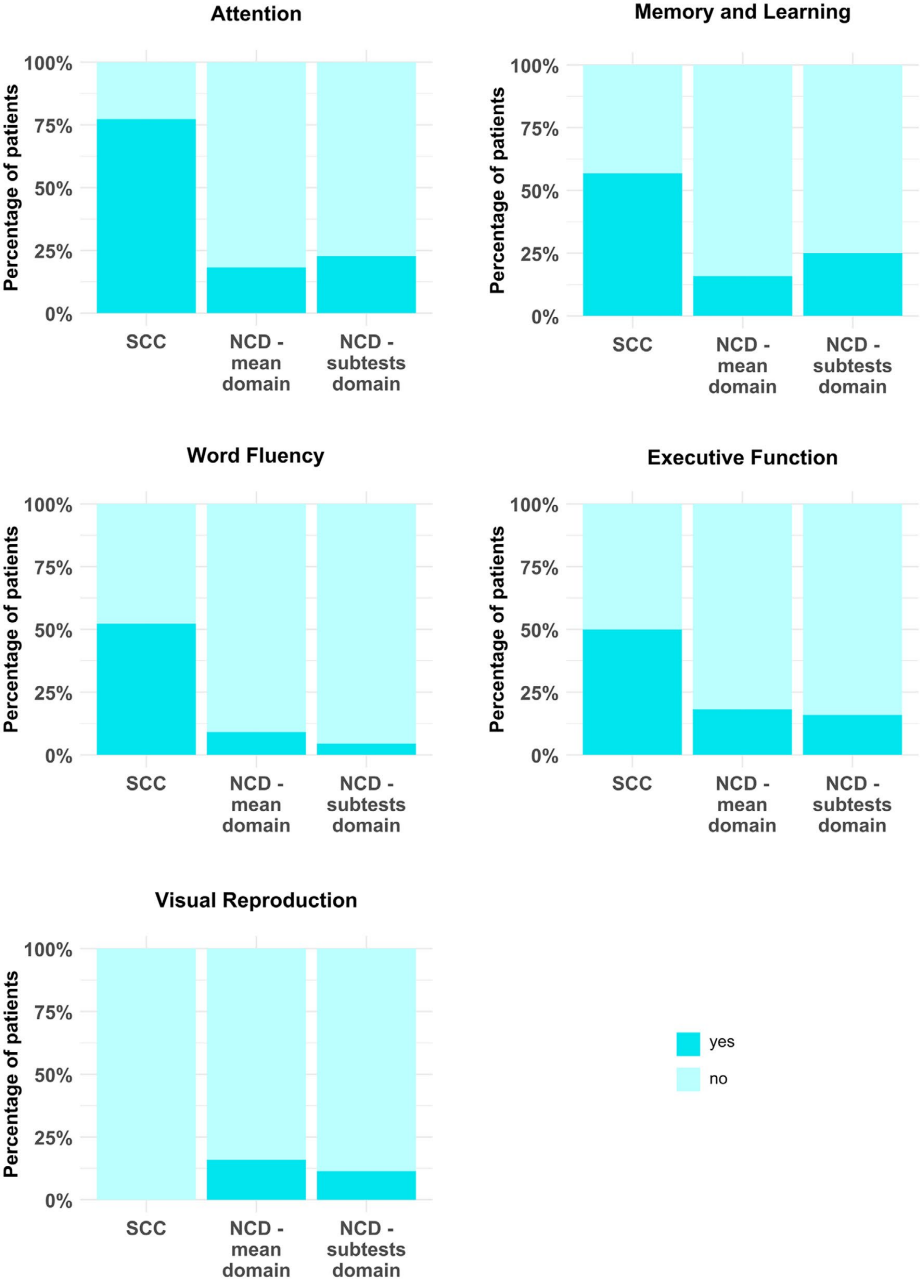
Subjective cognitive complaints (SCC) and objectively measured neurocognitive disorders (NCD) in the patient group without hospitalization (*N* = 44). The barplots show the percentages of patients who reported SCC in the respective cognitive domain as well as the percentages of patients who showed objective NCD with respect to the criteria “*mean domain*” and “*subtests domain*”. Mild and major NCD have been combined. (Note that the criterion “*subtests overall*” could not be illustrated in this figure since it did not take into account each domain separately.) No patient reported deficits in the domain *Visual Reproduction*.

Most patients from the group without hospitalization reported SCC in the domain of *Attention*, followed by *Memory and Learning*, *Word Fluency*, and *Executive Function*, while none reported symptoms or deficits in *Visual Reproduction* (Figure 1). In addition to complaints in these five cognitive domains, 45.5% of the patient sample reported headaches and 93.2% “rapid exhaustion” and/or “tiredness” in everyday life.

#### 3.1.3 Objective Neurocognitive Disorder (NCD)

Two criteria for objective NCD frequencies (“*mean domain*” and “*subtests domain*”) allowed to take into account each domain separately and thus could be illustrated in comparison with the patients' SCC (cf. Figure 1). Numerically, in the domains *Attention* and *Memory and Learning* slightly fewer patients with an NCD were identified using the “*mean domain*” criterion compared to “*subtests domain*” criterion. However, in the domains of *Executive Function*, *Word Fluency* as well as *Visual Reproduction*, the “*mean domain*” criterion identified a slightly higher number of affected patients. Detailed neuropsychological (sub‐)test results for each domain according to all three criteria are provided in Table S1 in the supplement.

To compare the effects of the different NCD cut‐off criteria on the NCD prevalence rate, we analyzed the same data set with respect to three different criteria. With the first and most liberal criterion (two subtests over all domains [“*subtests overall*”]) 24 patients (54.5%) were classified having mild and three patients (6.8%) having major NCD, while 17 (38.6%) had no NCD. Using the second criterion (mean *z*‐scores per domain [“*mean domain*”]), 14 patients (31.8%) were classified with mild and five patients (11.4%) with major NCD, while 25 patients (56.8%) had no NCD. Finally, using the third criterion by looking at two subtests per domain [“*subtests domain*”], 19 patients (43.2%) reached the cut‐off for mild and one patient (2.3%) for major NCD, while 24 (54.5%) had no NCD. Statistical comparison of the three different NCD cut‐off criteria revealed that there was no significant difference in terms of the proportion of patients with NCD (*χ* ^2^ [2] = 3.45, *p* = 0.178; *φ* = 0.28).

**FIGURE 2:**
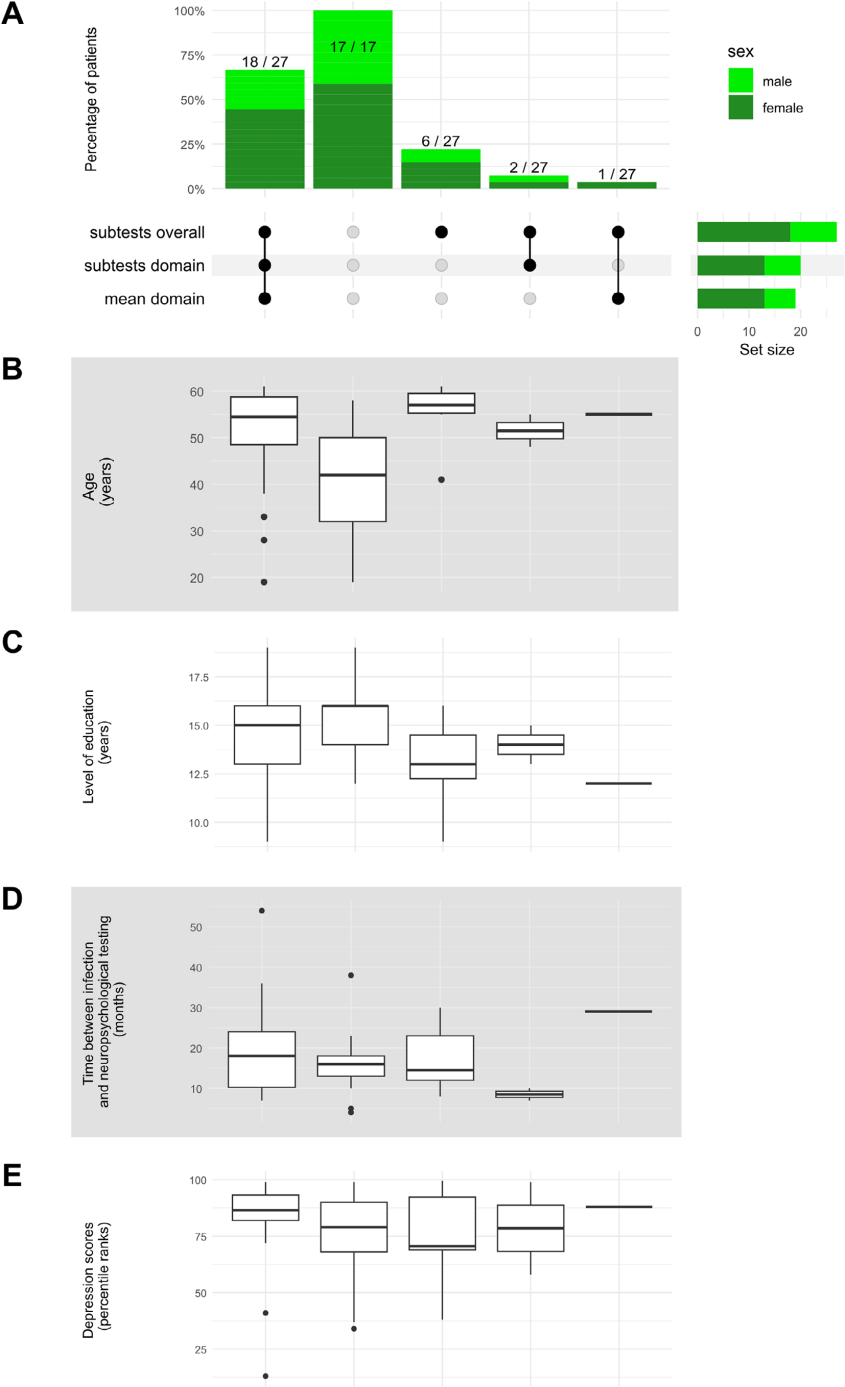
Comparison of the three neurocognitive disorder (NCD) cut‐off criteria “*subtests overall*”, “*subtests domain*” and “*mean domain*” using a complex UpSet plot. Mild and major NCD have been combined. (A) Sex: The UpSet plot shows the percentage of patients for each combination of NCD cut‐off criteria, broken down by sex. The lower right corner shows the number of patients per criterion who were classified as having a NCD. (B) Age: Boxplots with the median age of the respective patient group for each combination of NCD cut‐off criteria. (C) Level of education: Boxplots with the median level of education of the respective patient group for each combination of NCD cut‐off criteria. The level of education has been measured in school years plus years of study or vocational training. (D) Time between COVID‐19 infection and neuropsychological testing: Boxplots with the median time interval in months between the COVID‐19 infection and the neuropsychological evaluation of the respective patient group for each combination of NCD cut‐off criteria. (E) Depression: Boxplots with the median depression scores, illustrated in percentile ranks, of the respective patient group for each combination of NCD cut‐off criteria.

Figure 2 illustrates all demographic variables for the group of non‐hospitalized patients with and without objective NCD for each combination of the three NCD cut‐off criteria. All detailed demographic information can be found in the Table S2. The duration of education for this group, measured in school years plus years of study or vocational training, was 14.5 years (SD = 2.3, range 9–19). The patients' depression scores were above average, quantified in percentile ranks with a median of 85 (IQR = 21.3).

For the group of patients with NCD (in either of the three criteria), sex did not differ significantly between “*subtests overall*”, “*mean domain*” and “*subtests domain*” (*χ* ^2^ [2] = 0.05, *p* = 0.975; *φ* = 0.03). Also, age did not differ significantly between the three criteria (*χ* ^2^ [2] = 0.25, *p* = 0.883; *η* ^2^ = −0.03), nor did the time between COVID‐19 infection and neuropsychological examination (*χ* ^2^ [2] = 0.33, *p* = 0.849; *η* ^2^ = −0.03), the level of education (*χ* ^2^ [2] = 0.45, *p* = 0.797; *η* ^2^ = −0.02), or the depression scores (*χ* ^2^ [2] = 0.06, *p* = 0.969; *η* ^2^ = −0.03). For the group of patients without NCD, sex also did not differ significantly between “*subtests overall*”, “*mean domain*” and “*subtests domain*” (*χ* ^2^ [2] = 0.06, *p* = 0.969; *φ* = 0.04); also, age did not differ between the three criteria (*χ* ^2^ [2] = 1.97, *p* = 0.374; *η* ^2^ = −0.00), as well as the time between COVID‐19 infection and neuropsychological examination (*χ* ^2^ [2] = 0.33, *p* = 0.850; *η* ^2^ = −0.03), the level of education (*χ* ^2^ [2] = 1.24, *p* = 0.539; *η* ^2^ = −0.01) or depression scores (*χ* ^2^ [2] = 0.01, *p* = 0.996; *η* ^2^ = −0.03).

#### 3.1.4 Comparing SCC and NCD

**FIGURE 3:**
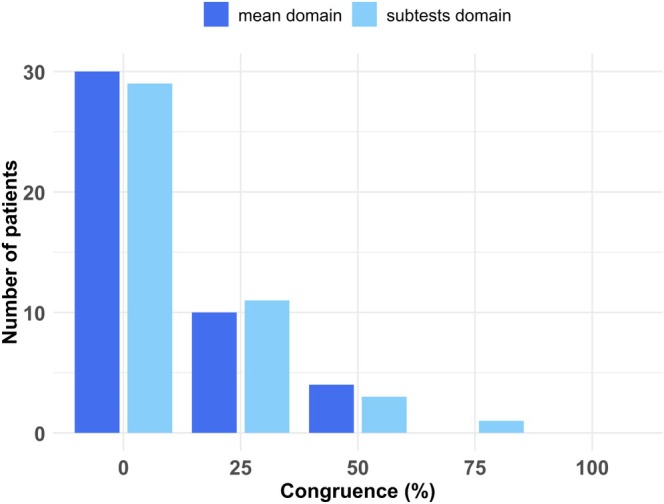
Global congruence between subjective cognitive complaints (SCC) and objective neurocognitive disorders (NCD) over four cognitive domains (without *Visual Reproduction* since no patient reported SCC in this domain). The barplot illustrates the number of patients who obtained different congruence percentages (measured as the average of congruence between four cognitive domains), separately for our NCD cut‐off criteria *mean domain* and *subtests domain*.

To investigate how well the objectively assessed cognitive deficits matched the subjectively reported cognitive complaints, we determined whether or not the subjectively reported deficit of a patient fitted with the objectively measured deficit (or non‐existent deficit) of the same patient. To this end, we measured the congruence on a global level, meaning that we looked at the congruence of a patient for each cognitive domain and then averaged it. As a result, we obtained global congruence percentages (50% congruency indicates that subjects had the same number of matches as non‐matches between SCC and NCD). Overall, the comparison between subjective complaints and objective findings did not show a high congruence (Figure 3). More than half of our patient sample was not congruent regarding SCC and NCD, that is, they had less than 50% congruency, and only one patient from our sample showed global congruence higher than 50% (Figure 3). In the non‐congruent or low‐congruent part of our patient sample (0% or 25% congruence), 66.67% were female. The mean age of all (male and female) patients in this subgroup was 47.1 years (SD = 11.9), the level of education 14.5 years (SD = 2.3) and the time between their COVID‐19 infection and the neuropsychological examination 18.2 months (SD = 10.1). Interestingly, the depression scores of all (male and female) patients in this subgroup were in the above‐average range (i.e., ≥ percentile rank of 84) in 51.3% of cases; the median depression score was a percentile rank of 86 (IQR = 24).

To go into more detail, we also determined the congruence between SCC and NCD separately for each cognitive domain (Table 2). In the majority of cases (between 60% and 95.65%, depending on the respective domain and criterion), the subjectively reported deficit in a specific cognitive domain could not be verified by the neuropsychological test results of this domain. The congruence between SCC and objective NCD was the highest in the domains *Memory and Learning* as well as *Attention* (cf. Table 2), while it was lowest in the cognitive domains *Executive Function* and *Word Fluency*.

**TABLE 2:** Domain‐specific congruence (in percent) of objective cognitive impairment (NCD) found in the non‐hospitalized patients (*N* = 44) who subjectively reported cognitive impairment (SCC) in that domain.

Cognitive domain	Congruence between SCC and NCD	
	Criterion “mean domain”	Criterion “subtests domain”
**Attention**, *N* = 34 reporting SCC	17.65%	17.65%
**Memory and Learning**, *N* = 25 reporting SCC	28.00%	40.00%
**Word Fluency**, *N* = 23 reporting SCC	8.69%	4.35%
**Executive Function**, *N* = 22 reporting SCC	13.64%	13.64%
**Visual Reproduction**, *N* = 0 reporting SCC	n.a.	n.a.

*Note:* In contrast to Figure 1, this table shows the domain‐specific congruence between subjective cognitive complaints (SCC) and neurocognitive disorder (NCD) for each individual; that is, we determined whether or not the subjectively reported deficit of a patient in one domain fitted with the objectively measured deficit (or non‐existent deficit) of the same patient in the same domain. Values are presented for only two of the three cut‐off criteria, since the criterion “*subtests overall*” is based on the number of impaired subtests from all subtests, regardless of domain. For the domain *Visual Reproduction* no congruence values can be provided because none of our patients reported symptoms or deficits in this domain.

Abbreviations: n.a., not applicable; NCD, objective neurocognitive disorder; SCC, subjective cognitive complaints.

On the other hand, if patients did not report SCC for a specific domain, this was consistent with our objective results in the majority of cases. However, some cases were also observed where the reverse was true. Patients who did not report deficits in the *Attention* domain had objective deficits in 40% (“*subtests domain*”) or 20% (“*mean domain*”). With regard to the domains *Memory and Learning* 5.3% (“*subtests domain*”) or 0% (“*mean domain*”), *Executive Function* 18.2% (“*subtests domain*”) or 22.7% (“*mean domain*”), and *Word Fluency* 4.8% (“*subtests domain*”) or 9.5% (“*mean domain*”) of patients who did not complain deficits had objective deficits in the corresponding domain.

### 3.2 COVID‐19 Infections That Required Hospitalization

Only two patients in our unselected sample that fulfilled the inclusion criteria (see above) had to be hospitalized during their acute COVID‐19 infection; one of them including a stay at the intensive care unit. Both patients reported SCC in only one cognitive domain each (patient 1: *Memory and Learning*, patient 2: *Word Fluency*). Furthermore, they reported “rapid exhaustion” and “tiredness” in everyday life but no headaches. Neither of the two hospitalized patients had a NCD for any of the three criteria (“*subtests overall*”, “*mean domain*”, “*subtests domain*”). Thus, there was no domain‐specific congruence of objectively measured (NCD) and subjectively reported (SCC) cognitive impairment in the two hospitalized patients. The duration of education of the two patients was 19 and 13 years; the depression percentile ranks 86 and 34 and thus above average in one case. Detailed neuropsychological (sub‐)test results are shown in Table S3 in the supplement.

## 4 Discussion

We investigated the congruence of subjective and objective cognitive deficits after a COVID‐19 infection in an unselected cohort of 46 patients with subjectively perceived cognitive complaints, successively admitted to our Center for Neurology over a period of a good four years. The cognitive domains that were subjectively complained about most frequently were attention and memory, while executive functions and language‐related abilities were complained about less. No patient complained about his/her visuo‐spatial abilities. We compared these subjective complaints with the objective, psychometric measurement of these abilities. In order to avoid any disagreement about how cognitive impairment should be assigned when objectifying these subjectively perceived cognitive impairments, we applied three different criteria, commonly used in the literature. As expected, the more liberal criterion (“*subtests overall*”) for defining NCD resulted in the highest rate of objective NCD, whereas the two more conservative criteria (“*mean domain*” and “*subtests domain*”) resulted in lower NCD rates. However, statistical comparison showed that the NCD rates in our sample did not differ significantly. Further, our results suggest that—independent of the classification criteria—SCC were only seldom in congruence with the objectively measurable NCD. In general, SCC rates were much higher compared to the objective NCD in all investigated cognitive domains. Only 9% of our non‐hospitalized patients had a congruence rate of ≥ 50% between their SCC report and the actually measured NCD; 91% showed either no congruence at all or one of only 25%.

Discrepancies between subjective and objective rates of cognitive impairment in PCS have also been found by other researchers. Klinkhammer et al. [38] observed that subjective “cognitive complaints […] exceed cognitive dysfunction by far”. Unfortunately, they did not provide exact percentages for the congruence between subjective complaints and objective deficits, as this comparison was not the focus of their study. Another study that investigated the congruence between self‐reported cognitive impairment and objective test results reported a small correlation (determined by linear regression analysis) between subjective and objective measures [39]. However, this study relied on only three neuropsychological tests rather than an entire test battery and, more important, did not aim to identify the proportion of patients who actually have objective cognitive deficits from those who reported subjective cognitive deficits. Comparable with the present study, Schild et al. [15] used comprehensive and domain‐specific neuropsychological tests and reported an overall correspondence between SCC and NCD of ~40%, when combining their baseline and follow‐up examinations. Our findings showed a similar congruence of maximally 40% (depending on the cognitive domain) between SCC and NCD. Despite the low congruence observed by our data as well as the data by Schild et al. [15], it is nevertheless important to recognize the number of individuals affected by PCS. Even if the percentage of patients with objective deficits is rather small, the impact on public health remains an important issue, as does the search for treatment options tailored to this patient group.

In addition to patients that subjectively complained about cognitive disorders that could not be objectified, we also observed patients who showed the opposite dissociation. These patients did not report SCC in a specific domain but showed objective results suggestive of a domain‐related NCD. The rates of patients with objective deficits but no subjective cognitive complaints ranged from 0% to 40%, depending on the cognitive domain. The question arises as to whether these patients simply forgot to mention deficits in these domains or whether they genuinely believe that they have no impairments in these domains. Since the procedure in our study did not include explicit asking about specific subjectively experienced deficits in each cognitive domain, it is difficult to determine the exact nature of this discrepancy. Our cohort of patients with subjectively perceived cognitive complaints was on average 47 years old, and their level of education was on average 14 years. Thus, our sample was very similar to the PCS patients with subjective and/or objective cognitive deficits investigated in previous studies [15, 19, 21]. In addition, our sample consisted almost exclusively of patients with COVID‐19 infections that did not require hospitalization in the acute phase, as in Fleischer et al. [40] Remarkably, we found no significant differences in the proportion of men and women in our sample, although previous studies have suggested a higher proportion of women in PCS patients (e.g., [21, 40, 41, 42, 43, 44]). The high level of education in our sample, with more than half having an academic degree or even a doctorate, is interesting and could possibly be explained by the fact that patients with a higher level of education may be more responsive to small cognitive changes or show a greater interest in health issues in general [45] and are more likely to present to hospitals [46]. In addition, the usual occupations in which highly educated patients work may require and consequently train (more) finely tuned cognitive resources [40]. These factors may explain why these patients are more attuned to their cognitive abilities and quicker to notice changes and subjective declines.

There is another important aspect that could underlie the present finding that the subjectively perceived cognitive deficits could not be measured objectively in the greater part of our unselected patient sample. Clearly, the depression scores of all (male and female) patients in the group who reported SCC but did not show an objective NCD were in the above‐average range in > 50% of cases. This level of depressive symptoms in our sample corresponds with previous observations of depressive symptoms or even clinical depression in PCS [43, 47, 48]. However, we would like to emphasize that our study does not allow us to draw conclusions about the causality of the depressive symptoms. On the one hand, it is possible that depressive symptoms or depression cause the subjective perception of a cognitive dysfunction and/or impairment [49, 50, 51, 52], particularly in the domains of executive function, memory, and attention, which were also most affected in our patient sample. Reduced cognitive abilities are even part of the DSM‐5 diagnostic criteria [37] for major depressive disorder. Consequently, depressive symptoms can impair both one's own cognitive abilities as well as their perception [53], regardless of the COVID‐19 infection. Schwert et al. [53] even showed that depressed patients underestimate their cognitive abilities, which, albeit in relation to a different disease, is congruent with the results of our present study. The same was also found in another study in which subjective and objective cognitive impairment following traumatic brain injury was investigated [54]. They also observed high levels of depression in their patient sample and interpreted their results primarily by an underestimation of cognitive performance. On the other hand, however, the observed depressive symptoms could be the consequence of the COVID‐19 infection itself, as depression and psychiatric diagnoses in general have an increased incidence after COVID‐19 [47, 55]. The causality between the experienced cognitive impairment in PCS and the presence of depressive symptoms therefore remains plausible in both directions.

## 5 Limitations

Perhaps, the most significant limitation is the lack of pre‐COVID‐19 neuropsychological test data, which prevents us from assessing the patients' cognitive status and depressive symptoms before their Covid‐19 infection. Additionally, our study would benefit from longitudinal data to track changes over time. The absence of any contrast groups constrains our ability to compare results and draw broader conclusions. Lastly, our sample with 46 patients finally included is relatively small, which may limit the generalizability of our results.

## 6 Conclusion

In conclusion, this study showed that subjective cognitive complaints following COVID‐19 infection often do not align with results from standardized neuropsychological tests. In our unselected sample, the vast majority of the patients showed either no congruence at all or one of only 25% between SCC and objectively measured NCD. The high rate of depressive symptoms in our patient sample might be one factor underlying this discrepancy. Addressing depressive symptoms in treatments and psychotherapy in PCS may alleviate the overall burden of suffering, ultimately leading to an improved quality of life for the affected patients.

## Author Contributions

**Sofia Wöhrstein:** conceptualization, formal analysis, data curation, visualization, project administration, writing – original draft, methodology, writing – review and editing. **Tamara Matuz:** conceptualization, investigation, writing – original draft, data curation, project administration, writing – review and editing. **Lilli Rötzer:** data curation, investigation, writing – review and editing. **Hans‐Otto Karnath:** conceptualization, writing – review and editing, methodology, supervision, funding acquisition, resources.

## Ethics Statement

This study was approved by the Ethics Committee of the Medical Faculty of the University of Tübingen, Germany (233/2024BO2) and performed in line with the principles of the Declaration of Helsinki (1964).

## Conflicts of Interest

The authors declare no conflicts of interest.

## Supporting information


**Table S1.** Mean *z*‐scores and standard deviations of all subtests of the neuropsychological evaluation for the group of non‐hospitalized patients (*N* = 44).
**Table S2.** Demographical variables for the non‐hospitalized patients with versus without objective neurocognitive disorder (NCD versus no NCD).
**Table S3.** Standardized *z*‐scores of all subtests of the neuropsychological evaluation for the two hospitalized patients.

## Data Availability

The data that support the findings of this study are available from the corresponding author upon reasonable request.
