# Supplementary material for: Post‐COVID‐Syndrome Patients Might Overestimate Own Cognitive Impairment
Source: Eur J Neurol. 2025 May 15;32(5):e70195. doi: 10.1111/ene.70195 (PMC12079761; doi:10.1111/ene.70195)
Supplement: Supplementary file 1 — Table S1. Mean z‐scores and standard deviations of all subtests of the neuropsychological evaluation for the group of non‐hospitalized patients (N = 44). Table S2. Demographical variables for the non‐hospitalized patients with versus without objective neurocognitive disorder (NCD versus no NCD). Table S3. Standardized z‐scores of all subtests of the neuropsychological evaluation for the two hospitalized patients. [file ENE-32-e70195-s001.docx]

**Supplementary Material**

**Post-COVID-Syndrome patients might overestimate own cognitive impairment**

Sofia Wöhrstein^1^, Tamara Matuz^1^, Lilli Rötzer^1^, Hans-Otto Karnath^1^

^1^ Center of Neurology, Division of Neuropsychology, Hertie Institute for Clinical Brain Research, University of Tübingen, Tübingen, Germany

**Supplementary Tables**

**Table S1.** **Mean z-scores and standard deviations of all subtests of the neuropsychological evaluation for the group of non-hospitalized patients (N = 44).**

| **Domain** | **Mean z-score (SD)** | **Cognitive task** | **Mean z-score (SD)** | **Neuropsychological (sub)test** |
| --- | --- | --- | --- | --- |
| **Attention** | -0.33 (0.56) | Divided attention | -0.51 (1.01) | TAP divided total |
|  |  | Tonic attention | -0.85 (1.15) | TAP tonic alertness |
|  |  | Phasic attention | -0.05 (1.10) | TAP phasic K value |
|  |  | Flexible attention ^N=40^ | 0.17 (0.82) | TAP flexible |
| **Memory and Learning** | -0.24 (0.60) | Verbal learning ^N=32^ | 0.25 (1.03) | VLMT sum rounds 1-5 /  WMS-IV logical memory |
|  |  | Verbal recall immediate | -0.45 (0.85) | VLMT difference round 5-round 6 /  WMS-IV logical memory |
|  |  | Verbal recall delayed | -0.51 (0.93) | VLMT difference round 5-round 7 /  WMS-IV logical memory |
|  |  | Verbal recognition | -0.20 (0.70) | VLMT recognition / WMS-IV logical memory |
|  |  | Short term memory | 0.00 (0.93) | WMS-IV digit span forward |
| **Executive Function** | 0.07 (0.53) | Working memory ^N=43^ | -0.05 (0.82) | NAI / WMS-IV digit span backward |
|  |  | Categorical switch word fluency | 0.22 (0.85) | RWT sports/fruits |
|  |  | Categorical switch word fluency | -0.32 (0.76) | RWT G/R-words |
|  |  | Cognitive planning ^N=37^ | 0.52 (1.06) | TL-D |
| **Word Fluency** | 0.02 (0.77) | Semantic word fluency | 0.28 (0.95) | RWT animals |
|  |  | Phonematic word fluency | -0.23 (0.90) | RWT P-words |
| **Visual Reproduction ^N=43^** | 0.55 (1.13) | Visual reproduction immediate ^N=43^ | 0.68 (1.21) | ROCFT / WMS-IV figure |
|  |  | Visual reproduction delayed ^N=43^ | 0.26 (1.40) | ROCFT / WMS-IV figure |
|  |  | Visual recognition ^N=17^ | -0.02 (0.57) | ROCFT / WMS-IV figure |

*Note*. Abbreviations: n.a. = not applicable; NAI = Nürnberg age inventory^1^; NCD = neurocognitive disorder; ROCFT = Rey-Osterrieth Complex Figure Test^2,3^; RWT = Regensburg word fluency test^4^; SD = standard deviation; TAP = attention test battery^5^; TL-D = Tower of London^6^; VLMT = verbal learning and memory ability test^7^; WMS-IV = Wechsler Memory Scale – Fourth Edition.^8^

**Table S2.** **Demographical variables for the non-hospitalized patients with versus without objective neurocognitive disorder (NCD versus no NCD).**

|  | **Criterion ‘*subtests overall*’** | | | **Criterion ‘*mean domain*’** | | | **Criterion ‘*subtests domain*’** | | |
| --- | --- | --- | --- | --- | --- | --- | --- | --- | --- |
|  | **No NCD** | **Minor NCD** | **Major NCD** | **No NCD** | **Minor NCD** | **Major NCD** | **No NCD** | **Minor NCD** | **Major NCD** |
| N | 17 | 24 | 3 | 25 | 14 | 5 | 24 | 19 | 1 |
| Age, *years* (SD) | 40.35  (11.81) | 50.79  (11.15) | 56.00 (5.29) | 44.80 (12.24) | 50.07 (12.75) | 50.40 (10.83) | 44.67 (12.48) | 50.05 (12.00) | 50.00 (n.a.) |
| Sex (f/m) | 10/7 | 16/8 | 2/1 | 15/10 | 9/5 | 4/1 | 15/9 | 12/7 | 1/0 |
| Months between infection and neuropsychological exam (SD) | 16.35 (7.61) | 17.96 (11.56) | 23.00 (3.61) | 15.96 (7.69) | 17.50 (9.57) | 26.80 (16.08) | 17.13 (7.91) | 17.95 (12.06) | 26.00 (n.a.) |
| Level of education, *years* (SD) | 15.12 (1.81) | 13.83 (2.35) | 16.67 (2.08) | 14.56 (2.10) | 14.21 (2.61) | 15.40 (2.30) | 14.50 (2.19) | 14.37 (2.24) | 19.00 (n.a.) |
| Depression scores, *PR median* (IQR) | 79  (22) | 86  (23) | 90.50 (8.5) | 79 (22) | 87 (12) | 87 (24.50) | 79 (21.25) | 86.50 (11.75) | n.a. |

*Note*. Abbreviations: SD = standard deviation; n.a. = not applicable; f = female; m = male; PR = percentile rank; IQR = interquartile range.

**Table S3.** **Standardized z-scores of all subtests of the neuropsychological evaluation for the two hospitalized patients.**

| **Domain** | **Patient 1** | **Patient 2** | **Cognitive task** | **Patient 1** | **Patient 2** | **Neuropsychological (sub)test** |
| --- | --- | --- | --- | --- | --- | --- |
| **Attention** | 0.28 | -0.70 | Divided attention | 0.00 | -1.28 | TAP divided total |
|  |  |  | Tonic attention | 0.71 | -1.18 | TAP tonic alertness |
|  |  |  | Phasic attention | 0.31 | -0.81 | TAP phasic K value |
|  |  |  | Flexible attention | 0.10 | 0.50 | TAP flexible |
| **Memory and Learning** | -1.00 | 0.74 | Verbal learning | -1.64 | 1.64 | VLMT sum rounds 1-5 / WMS-IV logical memory |
|  |  |  | Verbal recall immediate | -0.39 | 0.67 | VLMT difference round 5-round 6 /  WMS-IV logical memory |
|  |  |  | Verbal recall delayed | -0.39 | 0.52 | VLMT difference round 5-round 7 /  WMS-IV logical memory |
|  |  |  | Verbal recognition | -1.64 | 0.84 | VLMT recognition / WMS-IV logical memory |
|  |  |  | Short term memory | -0.92 | 0.00 | WMS-IV digit span forward |
| **Executive Function** | -0.82 | 0.35 | Working memory | -1.18 | 0.67 | NAI / WMS-IV digit span backward |
|  |  |  | Categorical switch word fluency | -0.10 | 0.61 | RWT sports/fruits |
|  |  |  | Categorical switch word fluency | -1.18 | 0.77 | RWT G/R-words |
|  |  |  | Cognitive planning ^N=1^ | n.a. | -0.67 | TL-D |
| **Word Fluency** | -0.70 | 0.50 | Semantic word fluency | -0.23 | 0.74 | RWT animals |
|  |  |  | Phonematic word fluency | -1.18 | 0.25 | RWT P-words |
| **Visual Reproduction** | 0.84 | 0.41 | Visual reproduction immediate | 0.50 | 0.41 | ROCFT / WMS-IV figure |
|  |  |  | Visual reproduction delayed | 1.18 | 0.41 | ROCFT / WMS-IV figure |
|  |  |  | Visual recognition ^a^ | n.a. | n.a. | ROCFT / WMS-IV figure |

*Note*. Abbreviations: n.a. = not applicable; NAI = Nürnberg age inventory^1^; NCD = neurocognitive disorder; ROCFT = Rey-Osterrieth Complex Figure Test^2,3^; RWT = Regensburg word fluency test^4^; SD = standard deviation; TAP = attention test battery^5^; TL-D = Tower of London^6^; VLMT = verbal learning and memory ability test^7^; WMS-IV = Wechsler Memory Scale – Fourth Edition^8^. ^a^This subtest was not performed on either of the two patients.

**References**

1. Oswald WD, Fleischmann UM. *Nürnberger-Alters-Inventar (NAI)*. 4. unveränderte Auflage ed. Hogrefe. Verlag für Psychologie; 1999.

2. Rey A. L'examen psychologique dans les cas d'encephalopathie traumatique. *Arch Psychol (Geneve)*. 1941;28:286-340.

3. Osterrieth PA. Le test de copie d'une figure complexe; contribution a l'etude de la perception et de la memoire. *Arch Psychol (Geneve)*. 1944;30:206-356.

4. Aschenbrenner S, Tucha O, Lange KW. *RWT: Regensburger Wortflüssigkeits-Test*. Hogrefe. Verlag für Psychologie; 2000.

5. *TAP - Testbatterie zur Aufmerksamkeitsprüfung*. Version 2.3.1. Psytest; 2007.

6. Tucha O, Lange KW. *TL-D. Turm von London - Deutsche Version*. Hogrefe. Verlag für Psychologie; 2004.

7. Helmstaedter C, Lendt M, Lux S. *Verbaler Lern-und Merkfähigkeitstest: VLMT*. Beltz-Test GmbH; 2001.

8. Lepach AC, Petermann F. Wechsler Memory Scale – Fourth Edition. Deutsche Bearbeitung. *Zeitschrift für Neuropsychologie*. 2012;
